# Antibiotic treatments to mothers during the perinatal period leaving hidden trouble on infants

**DOI:** 10.1007/s00431-022-04516-6

**Published:** 2022-06-10

**Authors:** Chenyang Ji, Geer Zhang, Siyuan Xu, Qingyi Xiang, Meishuang Huang, Meirong Zhao, Xiaoxia Bai

**Affiliations:** 1 Key Laboratory of Pollution Exposure and Health Intervention of Zhejiang Province, Interdisciplinary Research Academy, Zhejiang Shuren University, Hangzhou, 310015 China; 2 The Women’s Hospital, School of Medicine, Zhejiang University, Hangzhou, 310001 China; 3 Xixi Hospital of Hangzhou, Hangzhou, 310023 China; 4 Key Laboratory of Microbial Technology for Industrial Pollution Control of Zhejiang Province, College of Environment, Zhejiang University of Technology, Hangzhou, 310014 China

**Keywords:** Antibiotic treatments, Perinatal period, Infants, Gut microbiota, Antibiotic resistance

## Abstract

Antibiotic application during the perinatal period is unavoidable in the clinic, but the potential effects on mothers and infants remain unknown. Herein, 25 breast milk samples from mothers who received cefuroxime (CXM) or CXM + cefoxitin (CFX) treatments and fecal samples from their infants were collected to investigate the undesirable effects of antibiotics on the microbiota of mothers and neonates. Furthermore, five fecal samples of infants, whose mothers had antibiotic treatments, were collected at a 6-month postpartum follow-up visit to evaluate the long-term effects on infants’ gut microbiota. Moreover, the relative abundance of antibiotic resistance genes (ARGs) in fecal samples was compared to investigate the transfer of ARGs in the infant gut microbiota. The results indicated that the antibiotic treatments had no influence on the microbiota of breast milk. The dominant bacterial phyla in the fecal samples changed to *Firmicutes* and *Proteobacteria* after antibiotic treatments, while the bacterial community showed a recuperative trend at the follow-up visits. In addition, the abundance of ARGs in the infant gut microbiota demonstrated a declining trend in the CXM- and CXM + CFX-treated groups, while ARG abundance presented a significant increasing trend after a 6-month recovery period.

*Conclusion*: Antibiotic treatments for mothers during the perinatal period disturb the gut microbiota in neonates. The infants’ gut microbiota would partly return to their initial state after rehabilitation, but the transfer of ARGs would leave the hidden trouble of antibiotic resistance. Overall, the data presented here can help to guide the scientific use of antibiotics during the perinatal period and provide potential approaches to mitigate the negative consequences.

**Table:** 

**What is Known:** *• Antibiotic application during the perinatal period is unavoidable in the clinic.* *• Misuse of antibiotics can cause various unintended consequences, especially for antibiotic resistance.*	
**What is New:** *• Antibiotic treatments had no influence on the microbiota of breast milk but greatly disturbed the gut microbiota composition in infants.* *• The gut microbiota in infants would partly return to its initial state after rehabilitation but the transfer of ARGs would leave the hidden trouble of antibiotic resistance.*

**Supplementary information:**

The online version contains supplementary material available at 10.1007/s00431-022-04516-6.

## Introduction

Antibiotics are a large group of prescribed pharmaceuticals that are most commonly used to prevent and treat infectious diseases in humans, animals, and crops. Antibiotic usage on a global scale would certainly result in increasingly frequent detection of antibiotics in the environment. It has been reported that the highest antibiotic residue concentration in surface waters was up to 300 ng/L erythromycin [1]. The widespread use of antibiotics (the defined daily dose (DDD)/1000 inhabitants/day (DID) significantly increased from 18.71 in 2004 to 31.26 in 2016) has dramatically increased concerns about the negative effects on human gut microbiota [2]. Concerns about antibiotic residues in the environment arise from two aspects: the first is the potential threat of direct toxicity to human beings, and the second is whether low-level antibiotic exposure would result in microbial mutation with higher lethality or the possible development of resistant strains. Additionally, the transfer of antibiotic resistance genes (ARGs) from and between mutualists to pathogens would assist in the spread of antibiotic resistance [3, 4]. Accumulative evidence has revealed that the gut microbiota is involved in metabolism, pathogen resistance, immunomodulation, and even neural functions in the host [5]. Thus, a balanced compositional signature of gut microbiota is critical to the host’s health [6]. As a result, antibiotic-induced disturbance on the gut microbiota would definitely result in unintended effects on the host.

The rapid spread of antibiotic-resistant pathogens and incidental emergencies has drawn public attention to the issue of antibiotic resistance. Between 2014 and 2016, more than one million people died due to antibiotic-resistant microbial strain infections, and the death toll is anticipated to rise in the future [7]. Bacteria have developed various resistance mechanisms to withstand antibiotic exposure [8]. Except for mutational events, antibiotic resistance is most likely the result of lateral transfer of genes called ARGs from other bacteria [9]. Antibiotic-resistance selection may also occur within the gut microbiota, acting as a primary avenue for developing resistance in bacterial pathogens and subsequently transferring to pathogenic bacterial strains [3, 10]. Therefore, it is envisaged that the transfer of ARGs into the gut microbiota might weaken the efficacy of antibiotic treatments.

Clinically, antibiotics are often used throughout the perinatal period for treating urinary and bacterial vaginosis, as well as preventative measures during intrapartum/peripartum to reduce the risk of infection in the mother and newborn [11–13]. Under such circumstances, newborns are under great probability of antibiotic exposure. On the other hand, many previous studies have reported the presence of antibiotic residues in breast milk [14, 15]; thus, breastfeeding infants were exposed to antibiotics throughout the lactation phase. Human breast milk, which traditionally is considered sterile, has been discovered to provide a continual supply of commensal bacteria to the infant gut tract [16]. Furthermore, neonates are unavoidably subjected to antibiotic treatments due to their high vulnerability to bacterial infections during the neonatal period [17]. The composition of infants’ gut microbiota is also influenced by environmental factors such as diet, diseases, and antibiotic treatments [18]. However, the colonization of the sterile fetal gut tract by bacteria starts at birth when the neonate comes into contact with the vaginal and gut microbiota of the mother [19]. Perturbations to the gut microbiota, such as antibiotic applications, often result in altered colonization by various gut pathogens [20]. Contemplating all these, it is unsurprising that antibiotic treatments during parturition and the neonatal period have dramatic and long-lasting effects on infants’ gut microbiota. Moreover, the transfer of ARGs into the gut microbiota of infants would leave hidden trouble of antibiotic resistance. Applications of antibiotics during pregnancy have been previously connected with an increased incidence of food allergies, hay fever, and asthma in infants [21, 22]. However, little is known about the increasing risks of abnormal development in infants induced by maternal modification of the gut microbiota with clinical antibiotic treatments.

Herein, breast milk samples from mothers and fecal samples from neonates were collected and evaluated to probe the possible effects of antibiotic treatments on the normal microbiota of mothers and neonates. Additionally, fecal samples from infants whose mothers received antibiotics were collected at a 6-month postpartum follow-up visit to investigate the long-term effects of perinatal antibiotic treatments on the gut microbiota of infants. In addition, the abundance of ARGs in the fecal samples from different periods was also compared to analyze the transfer of ARGs in infant gut microbiota. The data presented here can shed light on how prenatal antibiotic treatments disrupt the homeostasis of gut microbiota and the development of antibiotic resistance in infants. All these factors could provide useful directions for clinical perinatal antibiotic applications.

## Material and methods

### Study population and sample collection

Breast milk and fecal samples were collected between May 7th and October 28th, 2020, at Women’s Hospital, School of Medicine, Zhejiang University. The inclusion criteria for the study were defined as those mothers who had no underlying health problems, psychological disorder, or trauma, were of childbearing age, did not receive antibiotics 3 months before parturition, and were not consuming probiotics 2 weeks before parturition. Similarly, those subjects who had a diagnosis or history of serious bacterial infectious disease (respiratory tract infection, intestinal infection, vaginal infection, and so on) during pregnancy, inability to breastfeed, and neonates with health problems at the time of birth were excluded from the study. Among the 25 participants, nine women who received no antibiotic treatment during the perinatal period were labeled as control group; 13 women who received cefuroxime (CXM; 1.5 g each time, and the defined daily doses were based on infection degree) were labeled as CXM-treated group; and three women who received CXM and cefoxitin (CFX; 1.5 g for CXM and 2 g for CFX each time, and the defined daily doses were based on infection degree) were labeled as CXM + CFX-treated group. After parturition, breast milk samples from mothers and the first fecal samples from neonates were collected. The follow-up visits were scheduled approximately 6 months after parturition before taking supplementary food. The inclusion criteria at this stage of the study were defined as those mothers who only received CXM antibiotic treatments during the perinatal period, those mothers who did not receive any antibiotic treatment or consumed any probiotics after parturition, those infants who were developing normally, and infants who were breastfed. Meanwhile, infants who either received antibiotics or probiotics, received any supplementary food, and had serious bacterial infectious disease (respiratory tract infection, intestinal infection, and so on) were excluded from the study. Five fecal samples of infants from CXM-treated mothers (CXM-FV) were collected at the follow-up visits. All breast milk and fecal samples were stored at −80 °C until further use. The breast milk samples were labeled as B-Con, B-CXM, and B-CXM + CFX groups based on antibiotic treatments given to mothers. Similarly, the fecal samples were labeled as F-Con, F-CXM, and F-CXM + CFX groups based on antibiotic treatments given to mothers; and fecal samples from follow-up visits were labeled as F-CXM-FV group.

### DNA extraction

The extraction of the global bacterial genome was conducted according to previous studies with slight modification [23]. Briefly, breast milk or fecal samples were thawed and suspended in 790 mL of sterile lysis buffer (4 M guanidine thiocyanate, 10% N-lauroyl sarcosine, 5% N-lauroyl sarcosine-0.1 M phosphate buffer) with the addition of 1 g of glass beads (0.1 mm, BioSpec Products, Inc., USA). The samples were subjected to vigorous vortexing, and the mixtures were then incubated for 1 h at 70 °C and beaten for 10 min at maximum speed. The bacterial DNA was extracted according to the manufacturer’s protocols using the E.Z.N.A.® Stool DNA Kit (Omega Bio-Tek, Inc., GA). The extracted DNA from each sample was used as the template for PCR amplification.

### PCR amplification and sequencing

To analyze the microbial community of breast milk and fecal samples, the V3-V4 regions of the bacterial 16S rRNA gene were amplified using universal primers supplemented with Illumina sequencing adapters and sample-specific barcodes according to Illumina’s instructions. The primer sequences of the universal primers (*16S rRNA*) are shown in Table S1. PCR reactions were run on an EasyCycler 96 PCR system (Analytik Jena Corp., AG). The PCR reaction system consisted of 1 mL of DNA template, 5 mL of 5 × *TransStart*® *FastPfu* buffer, 0.5 mL of 10 mM dNTPs, 0.5 mL of forward and reverse primers, 0.5μ L of *TransStart*® *FastPfu* DNA polymerase, and 17 mL of ddH_2_O. The PCR program was set as follows: denaturation at 95 ℃ for 3 min; 21 cycles of denaturation at 94 ℃ for 30 s, annealing at 58 ℃ for 30 s, elongation at 72 ℃ for 30 s; and extension at 72 ℃ for 5 min. The PCR products were indexed and mixed at equal ratios for Illumina sequencing by Shanghai Mobio Biomedical Technology Co. Ltd. using the MiSeq platform (Illumina Inc., USA) according to the manufacturer’s instructions.

To analyze the abundance of ARGs in fecal samples, the total DNA extractions were amplified using high-throughput quantitative PCR (HT-qPCR). The primer sequences of target ARGs are shown in Table S1. The HT-qPCR reaction was run on an Applied Biosystems ViiA TM 7 Real-Time PCR System (Wcgene Biotechnology, Shanghai). The PCR system consisted of 1 mL of DNA template, 5 mL of 2 × TB Green Premix Ex Taq II (Tli RNaseH Plus), 0.4 mL of forward and reverse primer, 0.2 mL of 50 × ROX reference dye, and 3 mL of ddH_2_O. The PCR program was set as follows: denaturation at 95 ℃ for 30 s; 40 cycles of denaturation at 95 ℃ for 5 s, annealing at 60 ℃ for 30 s. A final melting curve, ranging from 60 to 95 ℃, was then conducted to confirm the specificity of amplification.

### Data processing

Raw data from MiSeq sequencing were merged into one sequence based on the overlapping region of paired end reads. In addition, quality filtering for the raw reads and merged sequences was conducted according to the index and primer sequences on both ends of the sequences, and the sequence direction was corrected as well. Quality-filtered sequences were clustered into operational taxonomic units (OTUs) based on 97% similarity using Usearch (version 11, http://drive5.com/uparse/) and chimeric sequences were omitted in this step. The acquired representative OTU sequences were mapped with all optimized sequences to screen sequences with over 97% similarity of representative OTU sequences. To acquire the classified information of each OTU, representative OTU sequences with 97% similarity were subjected to taxonomic analysis based on Silva reference database (Release 138, http://www.arb-silva.de), and the microbiota composition was summarized at the phylum, class, order, family, genus, and species levels.

The richness and diversity of the microbial community were reflected by α-diversity indices using Mothur (version v.1.42.1, http://www.mothur.org/wiki/Schloss_SOP#Alpha_diversity). The microbial community richness was assessed using the Chao1 estimator (http://www.mothur.org/wiki/Chao) based on the following equation:

$${S}_{chao1}={S}_{obs}+\frac{{n}_{1}\left({n}_{1}-1\right)}{2\left({n}_{2}+1\right)}$$

where ${S}_{chao1}$ represents the estimated OTUs; ${S}_{obs}$ represents the observed OTUs; ${n}_{1}$ represents OTUs with one sequence; and ${n}_{2}$ represents OTUs with two sequences.

The microbial community diversity was assessed using the Shannon index (http://www.mothur.org/wiki/Shannon) based on the following equation:

$${H}_{\mathrm{shannon}}=-\sum_{i=1}^{{S}_{obs}}\frac{{n}_{i}}{N}\mathrm{ln}\frac{{n}_{i}}{N}$$

where ${S}_{obs}$ represents the observed OTUs; ${n}_{i}$ represents the sequence number of the *i*th OTU; and $N$ represents the total sequence number.

The comparisons of the relative abundance of the microbial community were performed using the rank-sum test. Comparisons between two independent groups were performed using a nonparametric Wilcoxon rank-sum test; comparisons of multiple groups were performed using a nonparametric Kruskal–Wallis rank-sum test. The differences in microbial community composition of multiple groups were reflected by b-diversity indices in QIIME. Principal coordinates analysis (PCoA) based on unweighted-UniFrac dissimilarity and permutational MANOVA (Adonis) were generated in the R package (version 3.6.0) vegan 2.5–7 using 10,000 permutations. The linear discriminant analysis (LDA) effect size (LEfSe) was used to detect taxa with differential abundance among groups (http://huttenhower.sph.harvard.edu/galaxy/root?tool_id=lefse_upload). Random forest conducted in QIIME (http://qiime.org/scripts/supervised_learning.html?highlight=random%20forest) was used to screen markedly different OTUs.

The relative abundances of ARGs were calculated using the method of DCt based on the following equation [24]:

$$F={2}^{-\Delta ({Ct}_{ARG}-{Ct}_{16S rRNA})}$$

where ${Ct}_{ARG}$ and ${Ct}_{16S rRNA}$ represent the threshold cycles of the target *ARG* and *16S rRNA* genes, respectively.

### Statistical analysis

R package (version 3.6.0) and SPSS (IBM Corp., NY, USA) were used for statistical analysis. The comparisons of the relative abundance of the microbial community were performed using the rank-sum test with the Wilcoxon rank-sum test for the comparisons of two independent groups and the Kruskal–Wallis rank-sum test for the comparisons of multiple independent groups. The comparisons of the relative abundance of ARGs were performed using one-way ANOVA with Tukey’s post hoc analysis. Statistical significance was set at *p < 0.05; **p < 0.01; ***p < 0.001.

## Results

### Overview of Illumina sequencing

In general, 55 samples (25 breast milk samples and 30 fecal samples) were sequenced using the MiSeq platform, and 1,482,182 optimized sequences were acquired after quality filtering. Overall, 161 OTUs were classified for the following analysis on the basis of 97% similarity.

### Microbiota in breast milk after perinatal antibiotic treatments

**Fig. 1:**
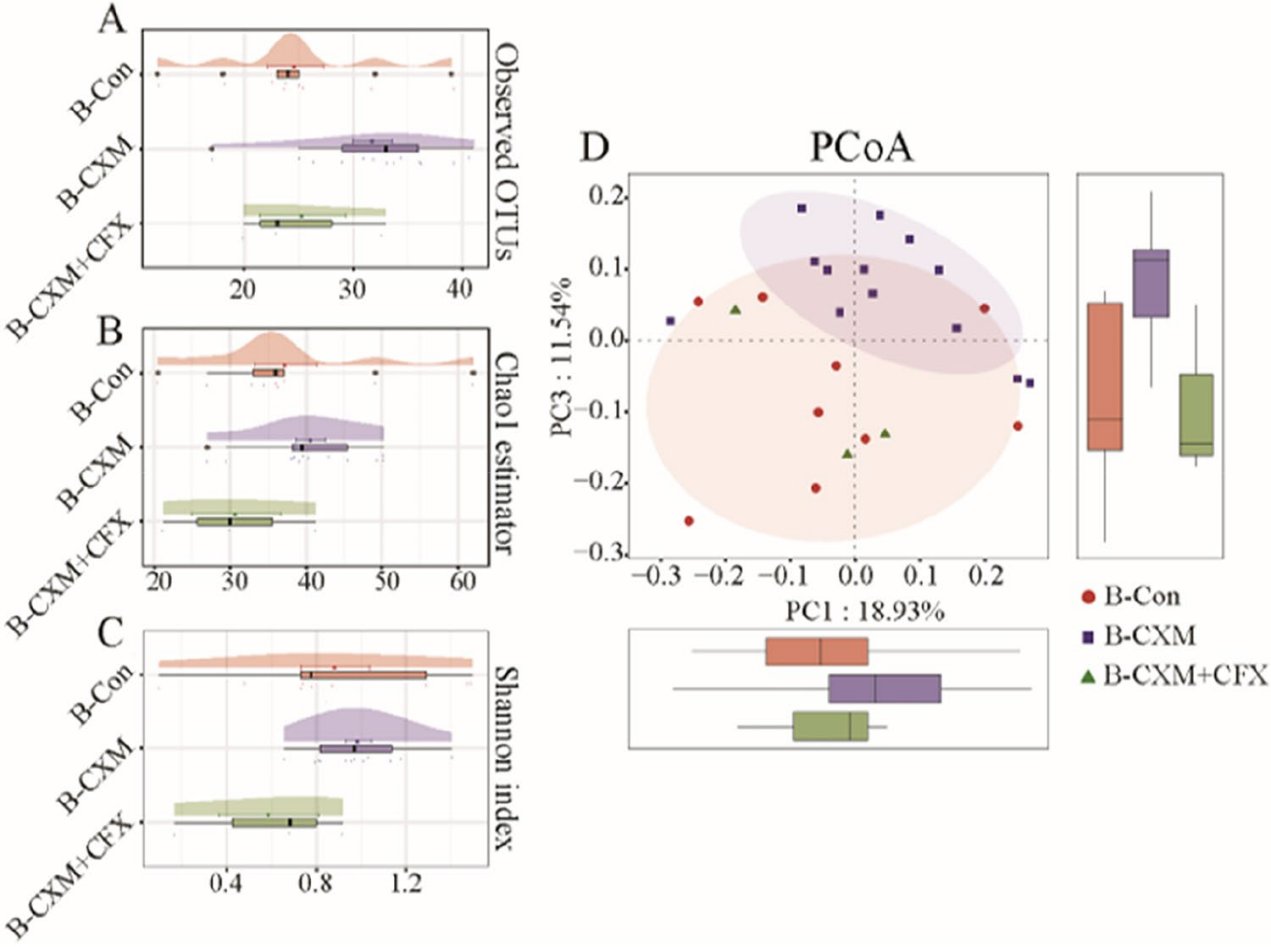
a and b-diversity indices of the microbiota in breast milk samples from the control group (B-Con, *n* = 9), CXM-treated group (B-CXM, *n* = 13), and CXM + CFX-treated group (B-CXM + CFX, *n* = 3). **A** Observed OTUs of the microbiota in breast milk samples; **B** cloud plot of the Chao1 estimator regarding the microbial community richness in breast milk samples; **C** cloud plot of the Shannon index regarding the microbial community diversity in breast milk samples; **D** multiple samples PCoA analysis regarding the difference in the microbial community composition in breast milk samples. Red circles represent samples of the B-Con group; purple squares represent samples of the B-CXM group; green triangles represent samples of the B-CXM + CFX group. Each box plot represents the median, interquartile range, minimum, and maximum values. *The data are statistically significantly different from the B-Con group (*p* < 0.05)

The disturbance to the microbiota in breast milk after perinatal antibiotic treatments was assessed based on the α-diversity indices. As illustrated in Fig. 1A–C, no significant difference in microbial community richness or diversity was observed in the breast milk samples from CXM-treated or CXM + CFX-treated mothers compared with the control group. According to the unweighted UniFrac dissimilarity-based PCoA scores (Fig. 1D), there was no significant difference in the microbial community composition between the control group and CXM-treated group (*p* > 0.05) according to the Adonis analysis.

**Fig. 2:**
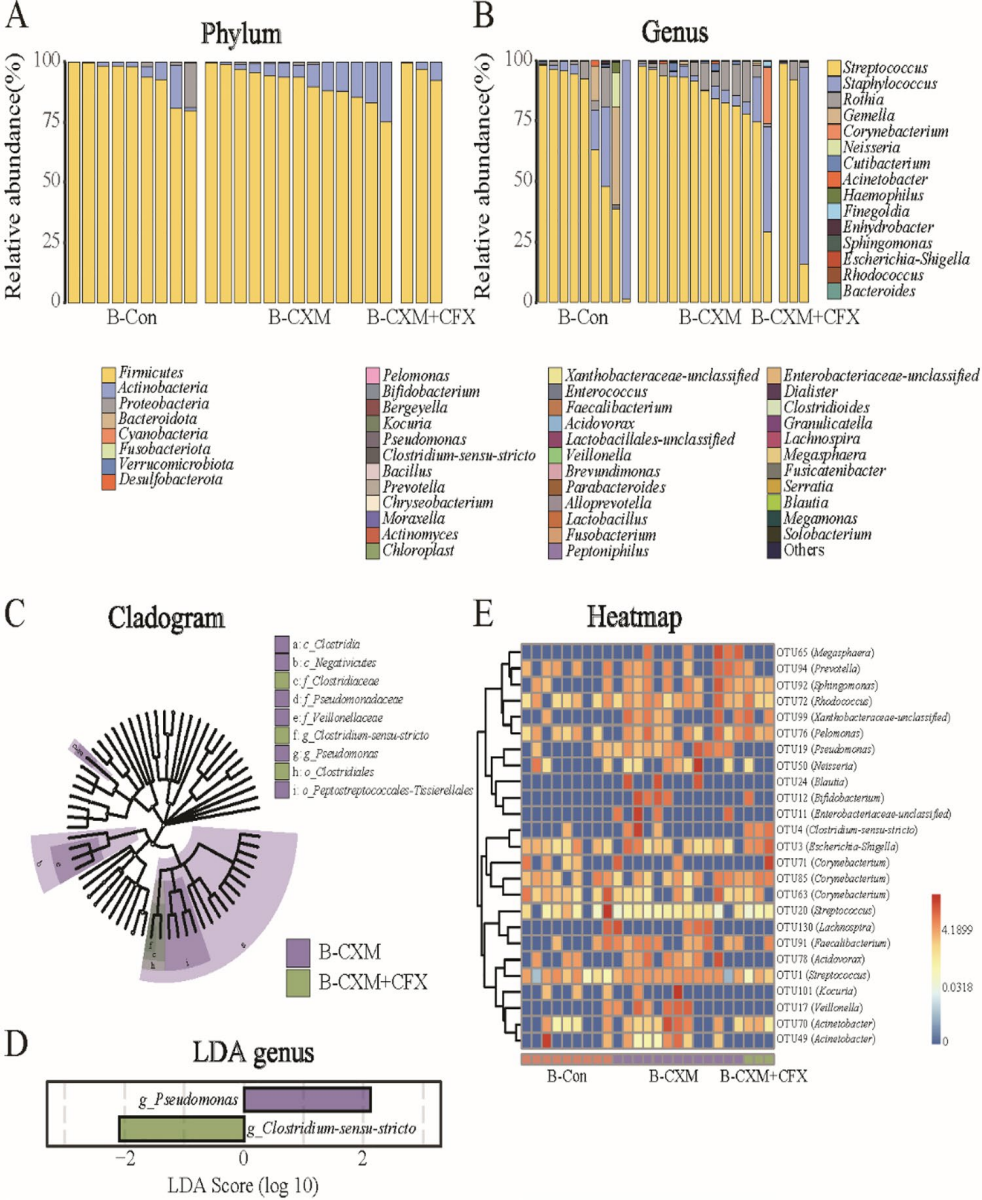
Comparison of the microbiota in breast milk samples from the B-Con group (*n* = 9), B-CXM group (*n* = 13), and B-CXM + CFX group (*n* = 3). **A** and **B** Microbial community bar plot of the microbiota in breast milk samples at the phylum and genus levels; **C** and **D** Kruskal–Wallis rank-sum test of the microbiota abundance in breast milk samples at the phylum and genus levels; **E** LEfSe analysis cladogram of distinct bacteria in breast milk samples at the phylum level; **F** LDA score of distinct bacteria in breast milk samples at the genus level; **G** microbial community heatmap regarding the microbiota abundance in breast milk samples. Red cells indicate increased; blue cells indicate decreased

The microbiome composition in breast milk at the phylum and genus levels across different groups is depicted in Fig. 2A and B. The bacterial community composition in breast milk was relatively simple, regardless of antibiotic treatments. *Firmicutes* were the most prevalent bacteria, followed by *Actinobacteria*. At the genus level, the most abundant bacterial genera were *Streptococcus*, *Staphylococcus*, and *Rothia*. The most vulnerable bacteria were identified by analyzing the differences in bacterial communities between groups. Generally, no dominate bacteria at the phylum or genus levels were identified in breast milk samples from the CXM- or CXM + CFX-treated groups when compared to the control group using the Kruskal–Wallis rank-sum test. LEfSe analysis reflected the significantly affected bacteria in breast milk (Fig. 2C and D). According to the LDA score at the genus level, the abundance of *Pseudomonas* was significantly higher in breast milk samples from the CFX-treated group (*p* < 0.05, LDA > 2), whereas *Clostridiaceae*-sensu-*stricto* was the most abundant bacteria in breast milk from CFX + CXM-treated group (*p* < 0.05, LDA > 2). Additionally, 25 significantly disturbed bacterial OTUs were identified in the microbiota of breast milk (Fig. 2E). In summary, CXM treatment upregulated 18 OTUs and downregulated 3 OTUs in breast milk microbiota compared with that of the control group. In breast milk from the CFX + CXM-treated group, 8 OTUs increased while 5 OTUs decreased compared with the control group.

### Gut microbiota in neonates

**Fig. 3:**
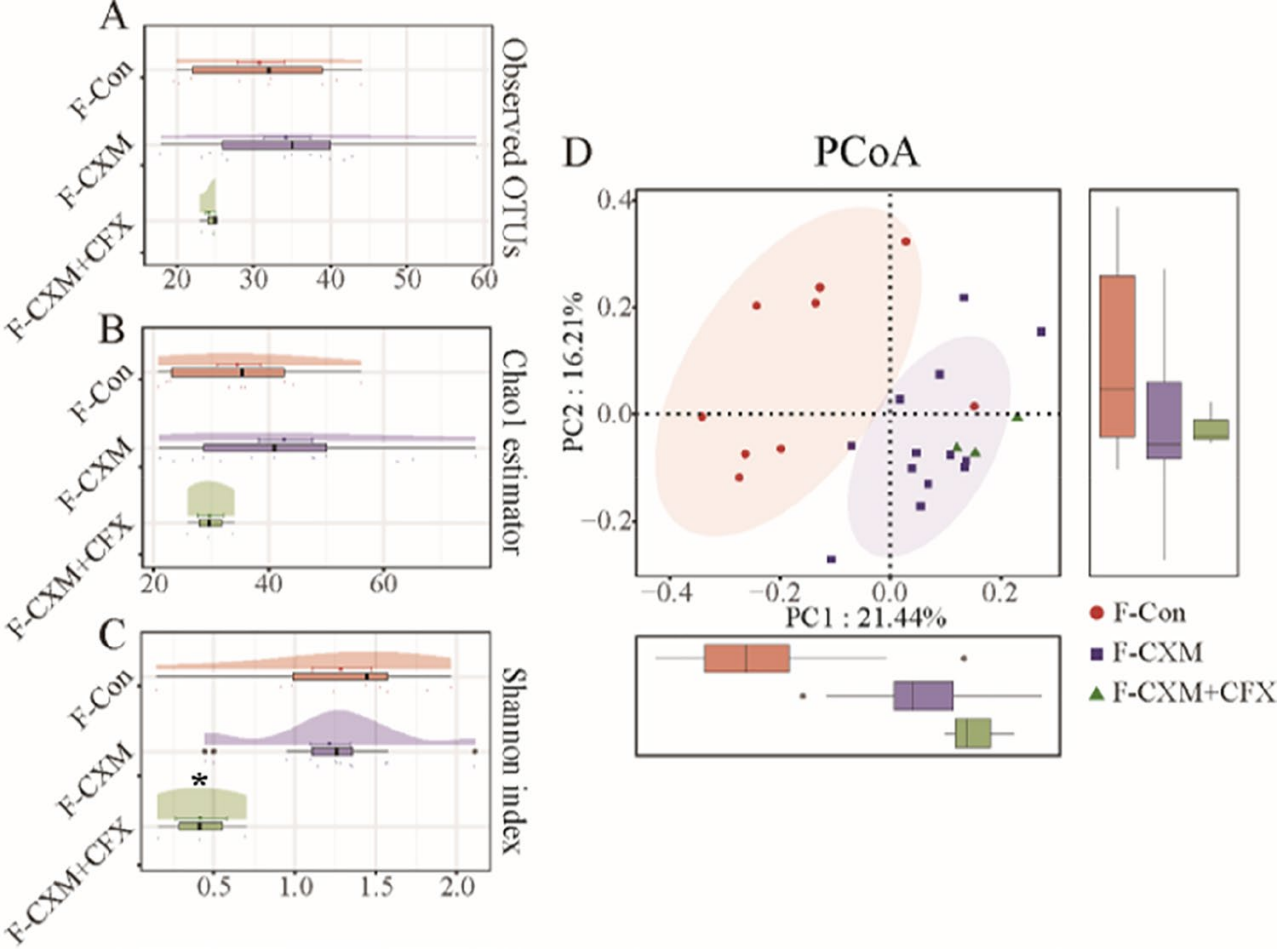
a and b-diversity indices of the gut microbiota in fecal samples from the control group (F-Con, *n* = 9), CXM-treated group (F-CXM, *n* = 13), and CXM + CFX-treated group (F-CXM + CFX, *n* = 3). **A** Observed OTUs in the gut microbiota in fecal samples; **B** cloud plot of the Chao1 estimator regarding the gut microbial community richness in fecal samples; **C** cloud plot of the Shannon index regarding the gut microbial community diversity in fecal samples; **D** multiple-samples PCoA analysis regarding the difference in the microbial community composition in fecal samples. Red circles represent samples of the F-Con group; purple squares represent samples of the F-CXM group; green triangles represent samples of the F-CXM + CFX group. Each box plot represents the median, interquartile range, minimum, and maximum values. *The data are statistically significantly different from the F-Con group (*p* < 0.05)

Antibiotic treatments to mothers during the perinatal period seemed to induce more profound effects on the gut microbiota in neonates. The Shannon index indicated no significant difference in the microbial community diversity in the fecal samples from the control group and CXM-treated group, but the microbial community diversity in the CXM + CFX-treated group was significantly lower than that in the control group and CXM-treated group. However, no significant difference was observed in the community richness of the gut microbiota in neonates from different groups (Fig. 3A–C). As shown in Fig. 3D, PCoA and Adonis analyses also revealed a huge discrepancy in the gut microbiota composition between the control group and the CXM + CFX-treated group (*p* < 0.001).

**Fig. 4:**
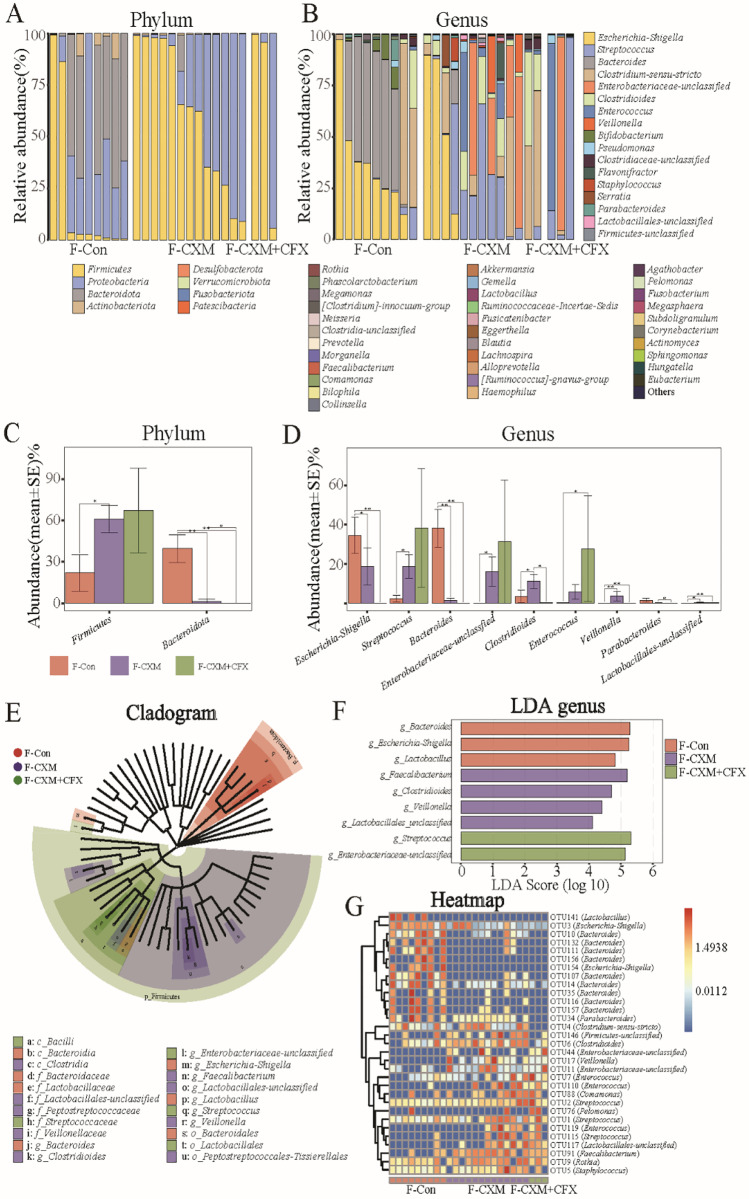
Comparison of the gut microbiota in fecal samples from the F-Con group (*n* = 9), F-CXM group (*n* = 13), and F-CXM + CFX group (*n* = 3). **A** and **B** Microbial community bar plot of the gut microbiota in fecal samples at the phylum and genus levels; **C** and **D** Kruskal–Wallis rank-sum test of the gut microbiota abundance in fecal samples at the phylum and genus levels; **E** LEfSe analysis cladogram of distinct bacteria in fecal samples at the phylum level; **F** LDA score of distinct bacteria in fecal samples at the genus level; **G** microbial community heatmap regarding the gut microbiota abundance in fecal samples. Red cells indicate increased; blue cells indicate decreased

Meanwhile, the dominant bacterial community in fecal samples was more abundant than that in breast milk samples (Fig. 4A and B). The most abundant bacterial phyla in the fecal samples from the control group followed the order of *Bacteroidota*, *Proteobacteria*, and *Firmicutes*. In the antibiotic treatment groups (both CXM and CXM + CFX treatments), the most dominant bacteria phylum was *Firmicutes* followed by *Proteobacteria*. At the genus level, the most abundant bacteria genera in fecal samples from the control group were *Bacteroides*, *Escherichia*-*Shigella*, and *Clostridium*-sensu-*stricto*. The dominant bacterial genera in the CXM-treated group were *Streptococcus*, *Clostridium*-sensu-*stricto*, *Escherichia*-*Shigella*, *Enterobacteriaceae*-*unclassified*, and *Clostridioides*. In CXM + CFX-treated group, the dominant bacterial genera were slightly different, which were *Streptococcus*, *Enterobacteriaceae*-*unclassified*, and *Enterococcus*. The Kruskal–Wallis rank-sum test reflected that CXM treatment enhanced the abundance of *Firmicutes* while decreasing *Bacteroidetes* abundance at the phylum level. At the genus level, CXM treatment reduced the abundance of *Bacteroides* and *Escherichia*-*Shigella* but increased the abundance of *Clostridioides*, *Enterobacteriaceae-unclassified*, *Lactobacillales-unclassified*, *Streptococcus*, and *Veillonella* (Fig. 4C and D). The significance in bacterial community differences of the CXM + CFX-treated group was subject to the limited clinical samples. As Fig. 4E and F shows, further LEfSe analysis revealed that *Bacteroides*, *Escherichia*-*Shigella*, and *Lactobacillus* were the most differently abundant bacterial genera in fecal samples from the control group (*p* < 0.05, LDA > 2). In the CXM-treated group, the most abundant bacterial genera were *Clostridioides*, *Faecalibacterium*, *Lactobacillales*-*unclassified*, and *Veillonella* (*p* < 0.05, LDA > 2). Regarding the CXM + CFX-treated group, the most susceptible bacterial genera were *Enterobacteriaceae*-*unclassified* and *Streptococcus* (*p* < 0.05, LDA > 2). In addition, the number of bacterial OTUs significantly disturbed by antibiotic treatments in fecal samples was up to 31 (Fig. 4G). Among these, OTU91 (*Faecalibacterium*), OTU19 (*Pseudomonas*), OTU17 (*Veillonella*), OTU4 (*Clostridiaceae*-sensu-*stricto*), OTU1 (*Streptococcus*), OTU76 (*Pelomonas*), and OTU11 (*Enterobacteriaceae*-*unclassified*) exhibited consistent phenotypes in breast milk and fecal samples.

### Gut microbiota alterations in infants

**Fig. 5:**
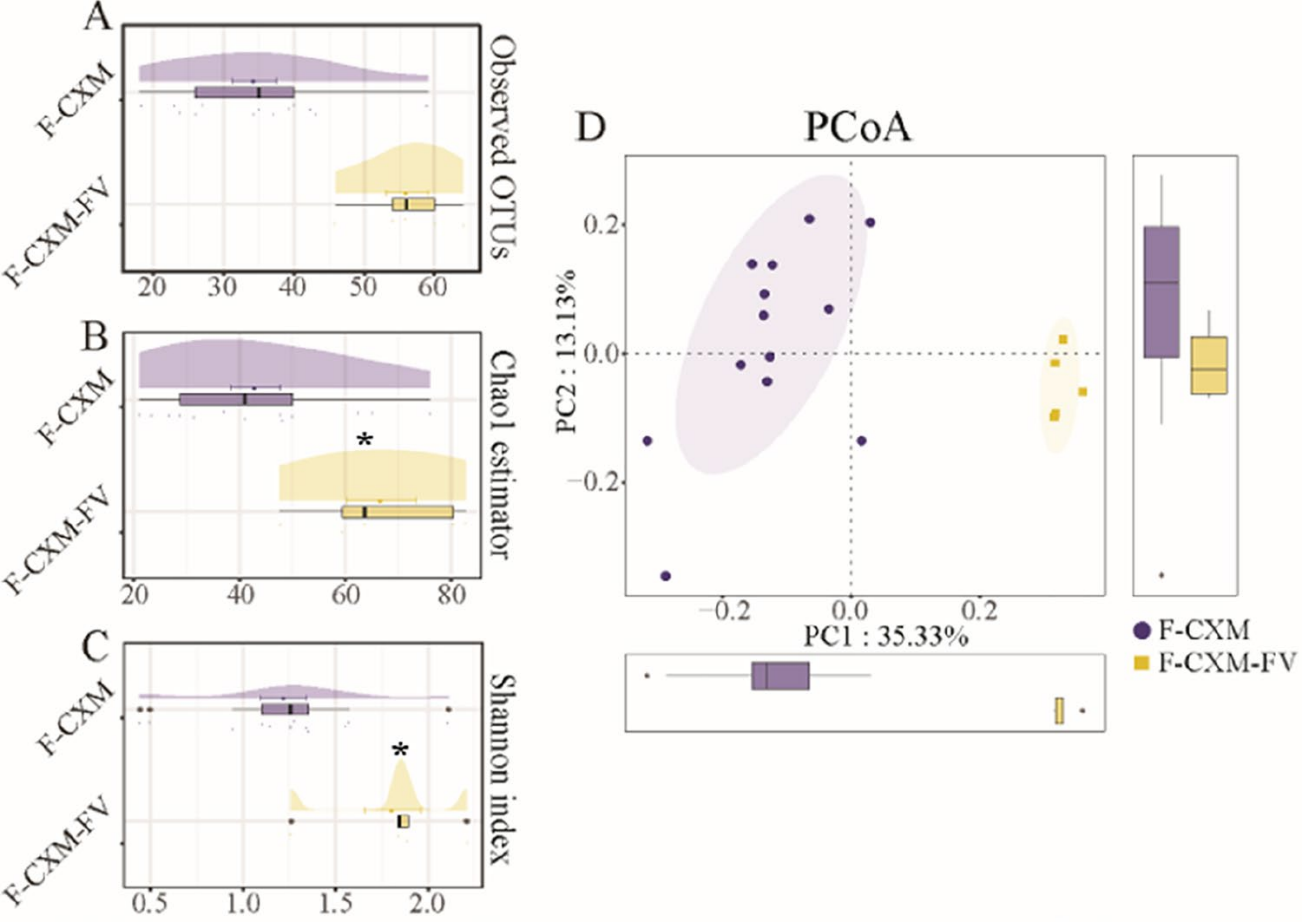
a and b-diversity indices of the gut microbiota in fecal samples from the CXM-treated group (F-CXM, *n* = 13) and CXM-treated group at the follow-up visits (F-CXM-FV, *n* = 5). **A** Observed OTUs in the gut microbiota in fecal samples; **B** cloud plot of the Chao1 estimator regarding the gut microbial community richness in fecal samples; **C** cloud plot of the Shannon index regarding the gut microbial community diversity in fecal samples; **D** multiple-samples PCoA analysis regarding the difference in the microbial community composition in fecal samples. Blue circles represent samples of the F-CXM group; yellow squares represent samples of the F-CXM-FV group. Each box plot represents the median, interquartile range, minimum, and maximum values. *The data are statistically significantly different from the F-CXM group (*p* < 0.05)

The gut microbiome composition in infants showed a substantial recuperative trend in the follow-up visits. The microbial community richness and diversity of the gut microbiota in the fecal samples of the CXM-FV group were significantly higher than those of the CXM-treated group (Fig. 5A–C). As illustrated in Fig. 5D, PCoA in conjunction with Adonis analysis revealed a significant change in the microbial community composition between the CXM-FV group and the CXM-treated group (*p* < 0.001).

**Fig. 6:**
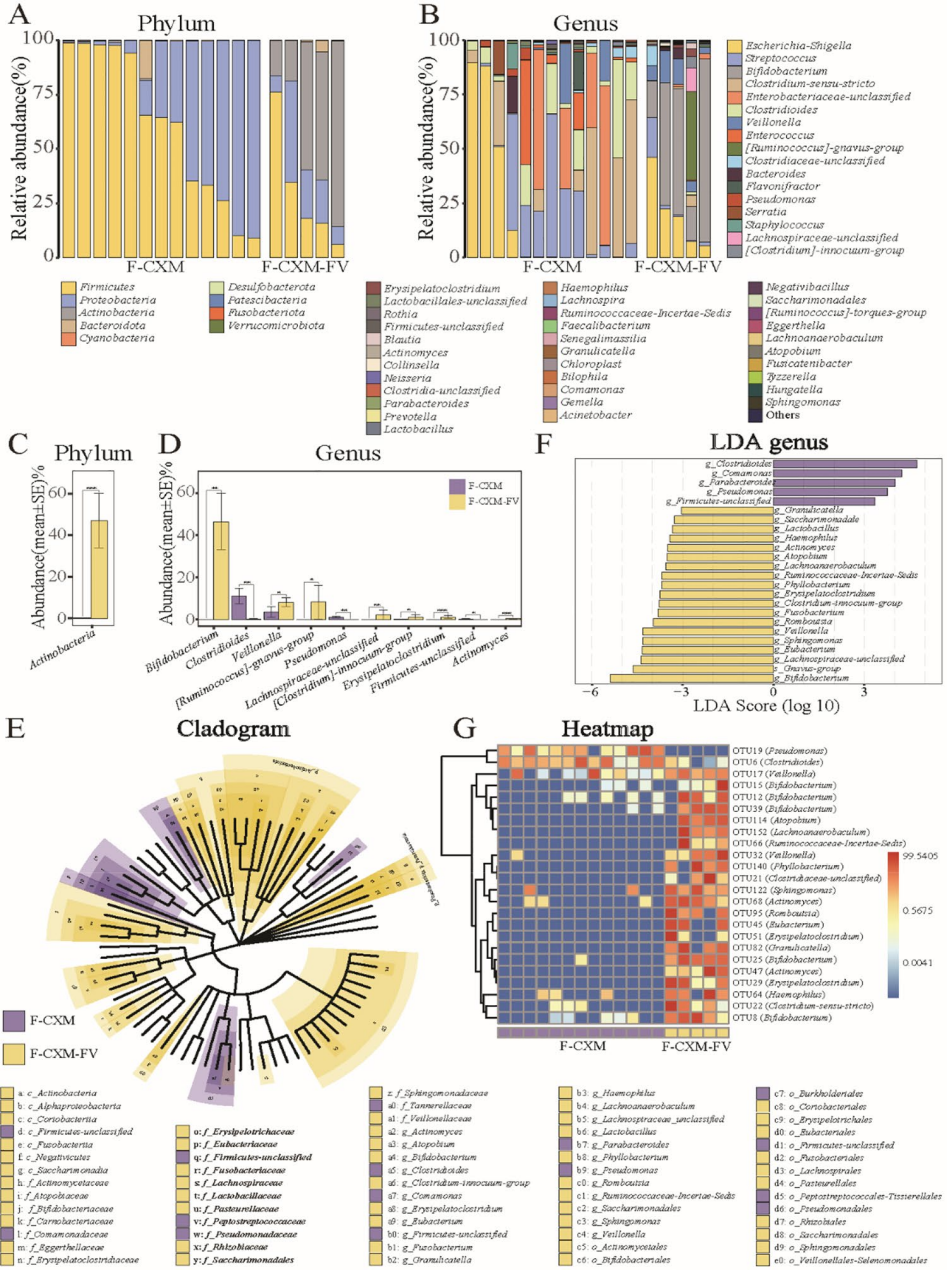
Comparison of the gut microbiota in fecal samples from the F-CXM group (*n* = 13) and F-CXM-FV group (*n* = 5). **A** and **B** Microbial community bar plot of the gut microbiota in fecal samples at the phylum and genus levels; **C** and **D** Wilcoxon rank-sum test of the gut microbiota abundance in fecal samples at the phylum and genus levels; **E** LEfSe analysis cladogram of distinct bacteria in fecal samples at the phylum level; **F** LDA score of distinct bacteria in fecal samples at the genus level; **G** microbial community heatmap regarding the gut microbiota abundance in fecal samples. Red cells indicate increased; blue cells indicate decrease

The dominant bacterial community in fecal samples of the CXM-FV group was also prone to revert to the initial state. The most abundant bacterial phyla in the fecal samples from the CXM-treated group were *Firmicutes* and *Proteobacteria*, while the dominant bacterial phyla in the CXM-FV group followed the order of *Actinobacteria*, *Firmicutes*, and *Proteobacteria* (Fig. 6A). At the genus level, *Escherichia*-*Shigella*, *Streptococcus*, *Clostridiaceae*-sensu-*stricto*, *Enterobacteriaceae-unclassified*, and *Clostridioides* were the most abundant bacterial genera in the CXM-treated group, whereas *Escherichia*-*Shigella* and *Bifidobacterium* were the most abundant bacterial genera in the CXM-FV group (Fig. 6B). The Wilcoxon rank-sum test revealed that *Actinobacteria* were considerably more abundant in the CXM-FV group at the phylum level. At the genus level, the CXM-FV group had a higher abundance of *Bifidobacterium* and another six bacterial genera, but the CXM-treated group had a lower abundance of *Clostridioides*, *Pseudomonas*, and *Firmicutes-unclassified* (Fig. 6C and D). As shown in Fig. 6E and F, LEfSe analysis further confirmed that *Clostridioides*, *Comamonas*, *Parabacteroides*, *Pseudomonas*, and *Firmicutes*-*unclassified* were the most distinct bacterial genera in the CXM-treated group (*p* < 0.05, LDA > 2), whereas *Bifidobacterium* and the remaining 18 bacterial genera were more abundant in the CXM-FV group (*p* < 0.05, LDA > 2). In addition, 24 significantly altered bacterial OTUs were identified between the CXM-treated group and the CXM-FV group, with only OTU19 (*Pseudomonas*) and OTU6 (*Clostridioides*) exhibiting lower abundance in the CXM-FV group (Fig. 6G).

### ARG abundance in the gut microbiota in neonates

**Fig. 7:**
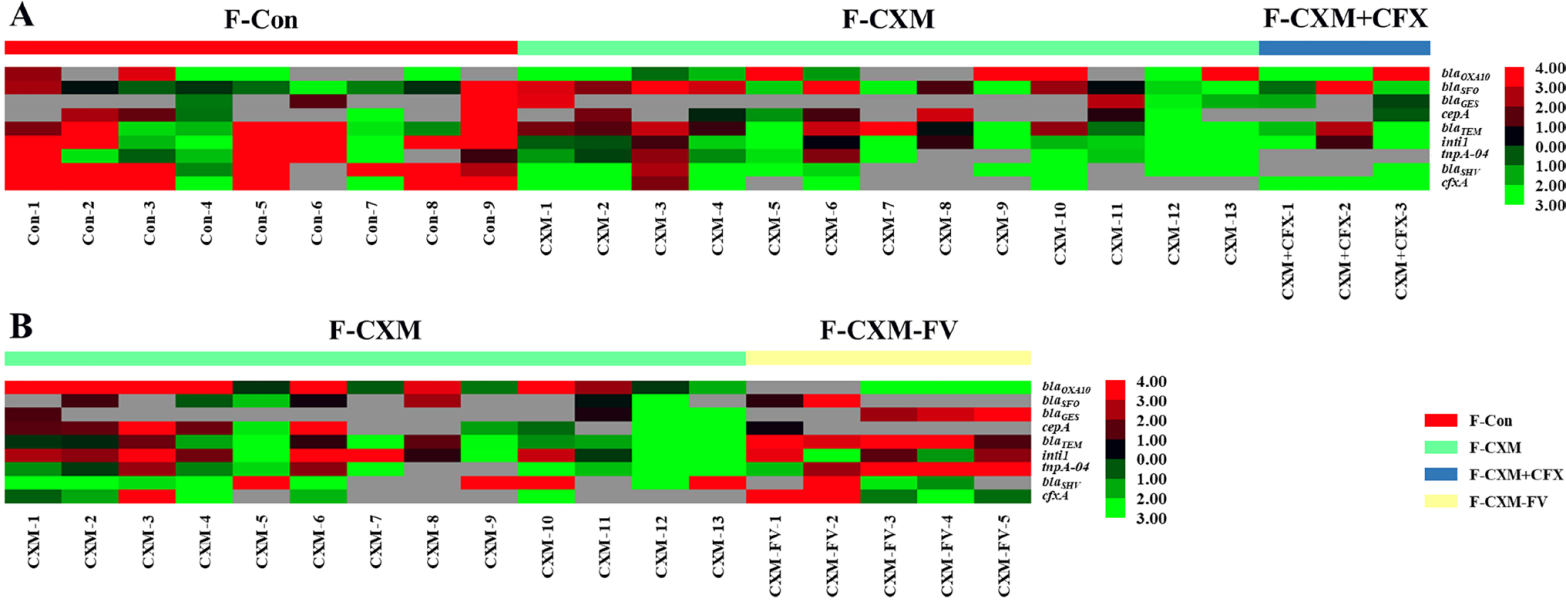
Heatmap regarding of the relative ARG abundance of the gut microbiota in fecal samples. **A** Relative ARG abundance of the gut microbiota in fecal samples from the F-Con group (*n* = 9), F-CXM group (*n* = 13), and F-CXM + CFX group (*n* = 3); **B** relative ARG abundance of the gut microbiota in fecal samples from the F-CXM group (*n* = 13) and F-CXM-FV group (*n* = 5). Red cells indicate increased; green cells indicate decreased

The ARG abundance in neonatal fecal samples was examined to evaluate antibiotic resistance in the gut microbiota. The results of HT-qPCR demonstrated that antibiotic treatments to mothers during the perinatal period made a difference to the antibiotic resistance in neonatal gut microbiota (Fig. 7A). The relative abundances of ARGs were substantially higher in fecal samples from the control group than those from the CXM-treated group. Over 50% of ARGs demonstrated declining trend in the CXM-treated group (particularly *bla*_*OXA10*_, *bla*_*SHV*_, *cfxA*, *tnpA-04*, and *tnti1*), while most ARGs were considerably downregulated in the CXM + CFX-treated group.

### ARG transfer in the gut microbiota of infants

ARG transfer in the gut microbiota of infants was investigated by comparing the obtained results with the fecal samples from the follow-up visits. As illustrated in Fig. 7B, the relative ARG abundance of the infants’ gut microbiota presented a significant increasing trend (*bla*_*GES*_, *bla*_*TEM*_, *tnpA-04*, and *tnti1*) after a 6-month recovery period.

## Discussion

Antibiotics are highly successful in controlling susceptible bacterial infections and undoubtedly provide remarkable benefits to human beings; however, the dark side of antibiotics also comes along with abuse. The excessive application of antibiotics not only caused the universal residue but also induced great disturbance to the microbial community in the host. Increasing evidence has confirmed that antibiotic treatments perturb the indigenous gut microbiota [5, 25, 26]. Therefore, there are increasing concerns regarding the unintended impacts of antibiotics on the human gut microbiota [27]. The unexpected outcomes of antibiotic abuse cover various aspects [26], among which, antibiotic resistance is the most worrisome issue that accounts for the lower efficacy of antibiotics as well as the development of resistant strains. Antibiotic treatments would alter the microbial community in breast milk, and antibiotics can also be transferred to the infants during lactation [28, 29]. Therefore, antibiotic treatments during the perinatal period could induce direct exposure to neonates as well as indirect effects via breast milk. More worryingly, transfer of ARGs in the gut microbiota of infants would leave the hidden trouble of antibiotic resistance in infants. Given all these findings, it is of great importance to investigate perinatal antibiotic treatment-induced effects on infants.

Breast milk, which is traditionally considered sterile, has been found to provide an ongoing supply of commensal bacteria to the infant gastrointestinal tract [16]. Previous studies also indicated that there was a transfer of bacterial strains, at least, to the genera of *Lactobacillus*, *Staphylococcus*, *Enterococcus*, and *Bifidobacterium*, belonging between mother and infant through lactation [30–33]. This is consistent with the most abundant bacterial phyla identified in breast milk from different groups (Fig. 2A). Hermansson et al. reported that perinatal antibiotic treatments were significantly associated with changes in milk microbial composition (*p* = 0.001), and higher breast milk microbiota α-diversity was considered to be associated with perinatal antibiotic treatments [34]. Thus, alteration of maternal breast milk microbiota during lactation could have a direct effect on infant health. The results of Illumina sequencing indicated that antibiotic treatments during the perinatal period had limited effects on the microbiota in breast milk. Only CXM + CFX treatment resulted in a significant disruption of the microbiota in breast milk, whereas CXM treatment showed a declining trend (*p* > 0.05). Regardless of the insignificant alteration in breast milk, the continuous disturbance to the gut microbiota of infants due to the long-term lactation period would leave unpredictable results.

On the other hand, antibiotics can be directly transferred to infants through breast milk. Existing studies have reported the detection of antibiotic residues in consumer milk, as well as other foods [35–37]. Smadi et al. reported an approximately 5% presence of antibiotic residues in breast milk samples from Syrian refugee nursing mothers (*n* = 120), who even had no antibiotic history [15]. Antibiotic residues in the mothers’ milk were believed to be a result of their daily diet [38]. Antibiotic ingestion by breast milk would definitely induce direct effects on the gut microbiota of infants. According to a previous study, administration of nonabsorbable antibiotics to pregnant dams altered the relative abundances of the *Lactobacillales* order and *Clostridium subcluster XVIII* family in antibiotic-treated dams as well as in the offspring [5]. Specific bacterial lineage blooms (*Akkermansia*, *Blautia*, *Enterococcus*, and *Faecalibacterium* genera) also occurred after antibiotic intervention [39]. Herein, the most dominant bacterial phylum in the antibiotic treatment groups (both CXM and CXM + CFX treatments) was *Firmicutes*, followed by *Proteobacteria*. Additionally, the consistent OTUs identified in breast milk and fecal samples (Figs. 2E and 4G) suggested that those bacterial OTUs could represent the critical microbiota correlated with antibiotic treatments. Mouse studies have indicated a causal role of early-life antibiotic use disrupting the microbiota with elevated risks for metabolic and immunological diseases [40]. Therefore, the hygiene hypothesis asserts that increasing rates of health issues in infants are related to the disruption of gut microbiota induced by antibiotics.

It is undoubted that antibiotic resistance has become a global problem due to diminished therapeutic effects and increased risks of complications and catastrophic outcomes [41]. ARGs are commonly regulated by sophisticated mechanisms that trigger gene transcription in response to antibiotic treatments [42]. CXM and CFX are typical cephalosporin-like antibiotics that are highly resistant to hydrolysis by β-lactamase [43, 44]; whereas *bla*_*TEM*_, *bla*_*SHV*_, *bla*_*OXA*_, and *bla*_*CTX-M*_ were identified as the genes encoding the extended-spectrum β-lactamase phenotype [45]. In this study, ARGs also exhibited a decreasing trend in the CXM-treated group (*bla*_*OXA10*_, *bla*_*SHV*_) and CXM + CFX-treated group (*bla*_*OXA10*_, *bla*_*SFO*_, *bla*_*TEM*_, *bla*_*SHV*_). These findings indicated that cephalosporin-like antibiotic treatments drastically disturb the gut microbiota in neonates but also introduce antibiotic resistance. Subirats et al. suggested that daily ingestion of antibiotics might expose the gut microbiota to antibiotic concentrations far exceeding the minimal selective concentration boundaries, which would favor the growth of potential resistant bacteria [46]. Thus, antibiotic treatments to mothers throughout the perinatal period would disturb the gut microbiota in infants by promoting resistant bacteria, and the disturbed gut microbiota would further impair infants’ health.

Although the microbiota can be affected by antibiotic treatments, little is known about their responses compared with baseline temporal variation. Measurements within individuals over time may reveal the range of variation conceivable in a system defined by the same set of interactions [25]. According to our findings, the diversity of gut microbiota in infants demonstrated a recuperative trend in follow-up visits after 6 months. Dethlefsen and Relman also reported that gut microbiota began to return to their initial states 1-week following antibiotic treatments, but the recovery was often incomplete [25]. As with other ecosystems, the gut microbiota at baseline is a dynamic system with a stable average state. Antibiotic treatments may cause a shift to an alternative stable state, but the full consequences remain unknown. In this study, the relative abundance of ARGs was dramatically elevated in follow-up visits, implying that ARGs were transferred to newborns’ gut microbiota. Among the varied biochemical mechanisms of antibiotic resistance, the acquisition of ARGs from the resistance gene pool of other microbial genera and antibiotic-producing organisms was believed to be the most likely resistance determinant [47, 48]. The transfer of ARGs from bacteria to human pathogens is bound to pose a threat to human health and public environmental sanitation. Given the observed transfer of ARGs in infant gut microbiota, antibiotic treatments to mothers during the perinatal period would definitely leave the hidden trouble of antibiotic resistance in their infants, therefore compromising therapeutic efficacy in the future.

In conclusion, our study found that antibiotic treatments to mothers during the perinatal period would disturb the gut microbiota in neonates by lactation. In addition, the gut microbiota in infants would partly return to their initial state after rehabilitation, but the transfer of ARGs in the gut microbiota of infants would leave hidden trouble of antibiotic resistance. We are aware that these results have been established with limited patients and will require further confirmation with a larger group of individuals and with other antibiotics. However, the data presented here can help to guide the scientific use of antibiotics during the perinatal period and provide viable approaches to mitigate the unanticipated consequences.

## Supplementary Information

Below is the link to the electronic supplementary material.

Supplementary file1 (DOCX 17 KB)

## Abbreviations

ARGs: Antibiotic resistance genes
B: Breast milk samples
CFX: Cefoxitin
Con: Control
CXM: Cefuroxime
DNA: Deoxyribonucleic acid
FV: Follow-up visits
F: Fecal samples
HT-qPCR: High-throughput quantitative PCR
LDA: Linear discriminant analysis
LEfSe: Linear discriminant analysis effect size
OTUs: Operational taxonomic units
PCoA: Principal coordinates analysis
PCR: Polymerase chain reaction
16S rRNA: 16S ribosomal RNA

## References

[1] Christian T, Schneider RJ, Frber HA, Skutlarek D, Meyer MT, Goldbach HE (2003). Determination of antibiotic residues in manure, soil, and surface waters. Clean.

[2] Lahoud N, Rizk R, Hleyhel M, Baaklini M, Zeidan R, Ajaka N, Rahme D, Maison P, Saleh N (2021). Trends in the consumption of antibiotics in the Lebanese community between 2004 and 2016. Int J Clin Pharm.

[3] Salyers AA, Gupta A, Wang Y (2004). Human intestinal bacteria as reservoirs for antibiotic resistance genes. Trends Microbiol.

[4] Sommer MOA, Dantas G, Church GM (2009). Functional characterization of the antibiotic resistance reservoir in the human microflora. Science.

[5] Tochitani S, Ikeno T, Ito T, Sakurai A, Yamauchi T, Hideo MH (2016). Administration of non-absorbable antibiotics to pregnant mice to perturb the maternal gut microbiota is associated with alterations in offspring behavior. PLoS ONE.

[6] Cryan JF, Dinan TG (2012). Mind-altering microorganisms: the impact of the gut microbiota on brain and behaviour. Nat Rev Neurosci.

[7] Ott A, Quintela-Baluja M, Zealand AM, O'Donnell G, Haniffah MRM, Graham DW (2021). Improved quantitative microbiome profiling for environmental antibiotic resistance surveillance. Environ Microbiome.

[8] Strauss C, Endimiani A, Perreten V (2015). A novel universal DNA labeling and amplification system for rapid microarray-based detection of 117 antibiotic resistance genes in Gram-positive bacteria. J Microbiol Meth.

[9] Aminov RI, Mackie RI (2010). Evolution and ecology of antibiotic resistance genes. FEMS Microbiol Lett.

[10] Chee-Sanford JC, Mackie RI, Koike S, Krapac IG, Lin YF, Yannarell AC, Maxwell S, Aminov RI (2009). Fate and transport of antibiotic residues and antibiotic resistance genes following land application of manure waste. J Environ Qual.

[11] Chan BT, Hohmann E, Barshak MB, Pukkila-Worley R (2013). Treatment of listeriosis in first trimester of pregnancy. Emerg Infect Dis.

[12] Hauth JC, Goldenberg RL, Andrews WW, Dubard MB, Copper RL (1995). Reduced incidence of preterm delivery with metronidazole and erythromycin in women with bacterial vaginosis. N Engl J Med.

[13] Turrentine MA, Greisinger AJ, Brown KS, Wehmanen OA, Mouzoon ME (2013). Duration of intrapartum antibiotics for group B streptococcus on the diagnosis of clinical neonatal sepsis. Infect Dis Obstet Gynecol.

[14] Dinleyici M, Yildirim GK, Aydemir O, Kaya TB, Bildirici Y, Carman KB (2018) Human milk antibiotic residue levels and their relationship with delivery mode, maternal antibiotic use and maternal dietary habits. Eur Rev Med Pharmaco 22:6560–6566. 10.26355/eurrev_201810_16072 10.26355/eurrev_201810_16072 30338827

[15] Smadi N, Jammoul A, Darra NE (2019). Assessment of antibiotic and pesticides residues in breast milk of Syrian refugee lactating mothers. Toxics.

[16] Fernández L, Langa S, Martín V, Maldonado A, Jiménez E, Martín R, Rodríguez JM (2013). The human milk microbiota: origin and potential roles in health and disease. Pharmacol Res.

[17] Noya F (1998). Antibiotic usage in neonates. Semin Pediatr Infect Dis.

[18] Tormo-Badia N, Håkansson Å, Vasudevan K, Molin G, Ahrné S, Cilio CM (2014). Antibiotic treatment of pregnant non-obese diabetic mice leads to altered gut microbiota and intestinal immunological changes in the offspring. Scand J Immunol.

[19] Fanaro S, Chierici R, Guenini P, Vigi V (2003). Intestinal microflora in early infancy: composition and development. Acta Paediatr.

[20] Sana TG, Monack DM (2016). Microbiology: the dark side of antibiotics. Nature.

[21] McKeever TM, Lewis SA, Smith C, Hubbard R (2002). The importance of prenatal exposures on the development of allergic disease: a birth cohort study using the West Midlands General Practice Database. Am J Respir Crit Care Med.

[22] Metsälä J, Lundqvist A, Virta LJ, Kaila M, Gissler M, Virtanen SM (2013). Mother’s and offspring’s use of antibiotics and infant allergy to cow’s milk. Epidemiology.

[23] Zhan QT, Qi XC, Weng RP, Xi FF, Chen Y, Wang YY, Hu W, Zhao BH, Luo Q (2021). Alterations of the human gut microbiota in intrahepatic cholestasis of pregnancy. Front Cell Infect Mi.

[24] Pu CJ, Liu H, Ding GC, Ying S, Yu XL, Chen JH, Ren JY, Gong XY (2017). Impact of direct application of biogas slurry and residue in fields: in situ analysis of antibiotic resistance genes from pig manure to fields. J Hazard Mater.

[25] Dethlefsen L, Relman DA (2011). Incomplete recovery and individualized responses of the human distal gut microbiota to repeated antibiotic perturbation. P Natl Acad Sci USA.

[26] Korpela K, De Vos W (2016) Antibiotic use in childhood alters the gut microbiota and predisposes to overweight. Microb Cell 3:296–298. 10.15698/mic2016.07.514 10.15698/mic2016.07.514 PMC5354595 28357367

[27] Blaser MJ, Falkow S (2009). What are the consequences of the disappearing human microbiota?. Nat Rev Microbiol.

[28] Lemas DJ, Yee S, Cacho N, Miller D, Cardel M, Gurka M, David JD, Shenkman E (2016). Exploring the contribution of maternal antibiotics and breastfeeding to development of the infant microbiome and pediatric obesity. Semin Fetal Neonatal Med.

[29] Taddio A, Ito S, Einarson TR, Leeder JS, Koren G (1995). Effect of counseling on maternal reporting of adverse effects in nursing infants exposed to antibiotics through breast milk. Reprod Toxicol.

[30] Jiménez E, Delgado S, Maldonado A, Arroyo R, Albújar M, García N, Jariod M, Fernández L, Gómez A, Rodríguez JM (2008). *Staphylococcus* epidermidis: a differential trait of the fecal microbiota of breast-fed infants. Bmc Microbiol.

[31] Martín R, Langa S, Reviriego C, Jimínez E, Marín M, Xaus J, Fernández L, Rodríguez JM (2003). Human milk is a source of lactic acid bacteria for the infant gut. J Pediatr.

[32] Martin R, Jimenez E, Heilig G, Fernandez L, Marin ML, Zoetendal EG, Rodriguez JM (2009). Isolation of *Bifidobacteria* from breast milk and assessment of the bifidobacterial population by PCR-denaturing gradient gel electrophoresis and quantitative real-time PCR. Appl Environ Microb.

[33] Martin V, Maldonado-Barragan A, Moles L, Rodriguez-Banos M, Campo RD, Fernandez L, Rodríguez JM, Jiménez E (2012). Sharing of bacterial strains between breast milk and infant feces. J Hum Lact.

[34] Hermansson H, Kumar H, Collado MC, Salminen S, Isolauri E, Rautava S (2019). Breast milk microbiota is shaped by mode of delivery and intrapartum antibiotic exposure. Front Nutr.

[35] Allison JRD (1985). Antibiotic residues in milk. Br Vet J.

[36] Baazize-Ammi D, Dechicha AS, Tassist A, Gharbi I, Hezil N, Kebbal S, Morsli W, Beldjoudi S, Saadaoui MR, Guetarni D (2019) Screening and quantification of antibiotic residues in broiler chicken meat and milk in the central region of Algeria. Rev Sci Tech 38:863–877. 10.20506/rst.38.3.3031 10.20506/rst.38.3.3031 32286562

[37] Nisha AR (2008). Antibiotic residues-a global health hazard. Vet World.

[38] Jammoul A, Darra EN (2019). Evaluation of antibiotics residues in chicken meat samples in Lebanon. Antibiotics.

[39] Ferrer M, Santos V, Ott SJ, Moya A (2013). Gut microbiota disturbance during antibiotic therapy: a multi-omic approach. Gut Microbes.

[40] Korpela K, Salonen A, Virta LJ, Kekkonen RA, Forslund K, Bork P, Vos WMD (2016). Intestinal microbiome is related to lifetime antibiotic use in Finnish pre-school children. Nat Commun.

[41] Andersson DI, Hughes D (2010). Antibiotic resistance and its cost: is it possible to reverse resistance?. Nat Rev Microbiol.

[42] Dar D, Sorek R (2017). Regulation of antibiotic-resistance by non-coding RNAs in bacteria. Curr Opin Microbiol.

[43] Onishi HR, Daoust DR, Zimmerman SB, Hendlin D, Stapley EO (1974). Cefoxitin, a semisynthetic cephamycin antibiotic: resistance to beta-lactamase inactivation. Antimicrob Agents Ch.

[44] Schumacher H, Skibsted U, Skov R, Scheibel J (1996). Cefuroxime resistance in Escherichia coli-resistance mechanisms and prevalence. APMIS.

[45] Literak I, Dolejska M, Janoszowska D, Hrusakova J, Meissner W, Rzyska H, Bzoma S, Cizek A (2010). Antibiotic-resistant Escherichia coli bacteria, including strains with genes encoding the extended-spectrum beta-lactamase and QnrS, in waterbirds on the Baltic Sea Coast of Poland. Appl Environ Microb.

[46] Subirats J, Domingues A, Topp E (2019). Does dietary consumption of antibiotics by humans promote antibiotic resistance in the gut microbiome?. J Food Protect.

[47] Neu HC (1992). The crisis in antibiotic resistance. Science.

[48] Silver LL, Bostian KA (1993). Discovery and development of new antibiotics: the problem of antibiotic resistance. Antimicrob Agents Ch.

