# Supplementary material for: Antibiotic treatments to mothers during the perinatal period leaving hidden trouble on infants
Source: Eur J Pediatr. 2022 Jun 10;181(9):3459–71. doi: 10.1007/s00431-022-04516-6 (PMC9395442; doi:10.1007/s00431-022-04516-6)
Supplement: Supplementary file 1 — Supplementary file1 (DOCX 17 KB) [file 431_2022_4516_MOESM1_ESM.docx]

**Supporting Information**

Antibiotic treatments to mothers during the perinatal period leaving hidden trouble on infants

Chenyang Ji^1^, Geer Zhang^2^, Siyuan Xu^2^, Qingyi Xiang^2^, Meishuang Huang^3^, Meirong Zhao^4^, and Xiaoxia Bai^2,*^

*1. Key Laboratory of Pollution Exposure and Health Intervention of Zhejiang Province, Interdisciplinary Research Academy, Zhejiang Shuren University, Hangzhou 310015*

*2. The Women’s Hospital, School of Medicine, Zhejiang University, Hangzhou, 310001*

*3. Xixi Hospital of Hangzhou, Hangzhou, 310023*

*4. Key Laboratory of Microbial Technology for Industrial Pollution Control of Zhejiang Province, College of Environment, Zhejiang University of Technology, Hangzhou, 310014*

^*^To whom correspondence should be addressed.

Email: [baixiaoxia@zju.edu.cn](mailto:baixiaoxia@zju.edu.cn); Address: No 1. Xueshi Road, Shangcheng District, Hangzhou, Zhejiang, 310001, China.

**Table S1**. Primer sequences of target genes.

| **Genes** | **Primer sequences (5’-3’)** |
| --- | --- |
| *16S rRNA* | F: CCTACGGGRSGCAGCAG  R: GGACTACVVGGGTATCTAATC |
| *bla_GES_* | F: GCAATGTGCTCAACGTTCAAG  R: GTGCCTGAGTCAATTCTTTCAAAG |
| *bla_OXA10_* | F: CGCAATTATCGGCCTAGAAACT  R: TTGGCTTTCCGTCCCATTT |
| *bla_SFO_* | F: CCGCCGCCATCCAGTA  R: GGGCCGCCAAGATGCT |
| *bla_SHV_* | F: CTTTCCCATGATGAGCACCTTT  R: TCCTGCTGGCGATAGTGGAT |
| *bla_TEM_* | F: AGCATCTTACGGATGGCATGA  R: TCCTCCGATCGTTGTCAGAAGT |
| *cepA* | F: AGTTGCGCAGAACAGTCCTCTT  R: TCGTATCTTGCCCGTCGATAAT |
| *cfxA* | F: TCATTCCTCGTTCAAGTTTTCAGA  R: TGCAGCACCAAGAGGAGATGT |
| *inti1* | F: CGAACGAGTGGCGGAGGGTG  R: TACCCGAGAGCTTGGCACCCA |
| *tnpA-04* | F: CCGATCACGGAAAGCTCAAG  R: GGCTCGCATGACTTCGAATC |
